# Recent Developments in Ozone Sensor Technology for Medical Applications

**DOI:** 10.3390/mi11060624

**Published:** 2020-06-26

**Authors:** Lisa Petani, Liane Koker, Janina Herrmann, Veit Hagenmeyer, Ulrich Gengenbach, Christian Pylatiuk

**Affiliations:** Institute for Automation and Applied Informatics, Karlsruhe Institute of Technology, 76344 Karlsruhe, Germany; liane.koker@kit.edu (L.K.); urgwh@student.kit.edu (J.H.); veit.hagenmeyer@kit.edu (V.H.); ulrich.gengenbach@kit.edu (U.G.); pylatiuk@kit.edu (C.P.)

**Keywords:** ozone sensors, medical applications, dissolved ozone, ozone gas, electrochemical sensors, optical sensors, inkjet-printing

## Abstract

There is increasing interest in the utilisation of medical gases, such as ozone, for the treatment of herniated disks, peripheral artery diseases, and chronic wounds, and for dentistry. Currently, the in situ measurement of the dissolved ozone concentration during the medical procedures in human bodily liquids and tissues is not possible. Further research is necessary to enable the integration of ozone sensors in medical and bioanalytical devices. In the present review, we report selected recent developments in ozone sensor technology (2016–2020). The sensors are subdivided into ozone gas sensors and dissolved ozone sensors. The focus thereby lies upon amperometric and impedimetric as well as optical measurement methods. The progress made in various areas—such as measurement temperature, measurement range, response time, and recovery time—is presented. As inkjet-printing is a new promising technology for embedding sensors in medical and bioanalytical devices, the present review includes a brief overview of the current approaches of inkjet-printed ozone sensors.

## 1. Introduction

Nowadays, treatment of chronic pain—such as low back pain, coronary artery disease, osteoarthritis, and peripheral artery disease—is a burden for society and the economy, and therefore highly relevant [1,2,3]. Gaskin et al. [4] estimate that the national cost of chronic pain treatment ranges in the United States from 560 to 635 billion dollars. Further research is necessary to successfully prevent and treat chronic pain [4].

Conventional treatment of pain caused by a herniated disk or osteoarthritis of the joints consists of repeated injections of anti-inflammatory drugs—such as corticosteroids, immunosuppressive drugs, and antibiotics [5,6]. These drugs are only partially and transiently effective. Furthermore, there are potential side effects—and in some cases they do not even relieve the pain.

This problem has led to novel and different treatment methods for pain therapy and related indications. Some of them are already well-established in clinical routine. For example, subcutaneous carbon dioxide is utilised to relieve muscle pain and nitrous oxide is applied as an anaesthetic in obstetrics and during prostrate examinations [7,8]. Furthermore, there are other gases with therapeutical potential, such as ozone [5,9,10,11,12,13,14] and xenon [15]. However, for xenon further research in clinical studies is necessary to prove its therapeutic effectiveness [15].

**Figure 1:**
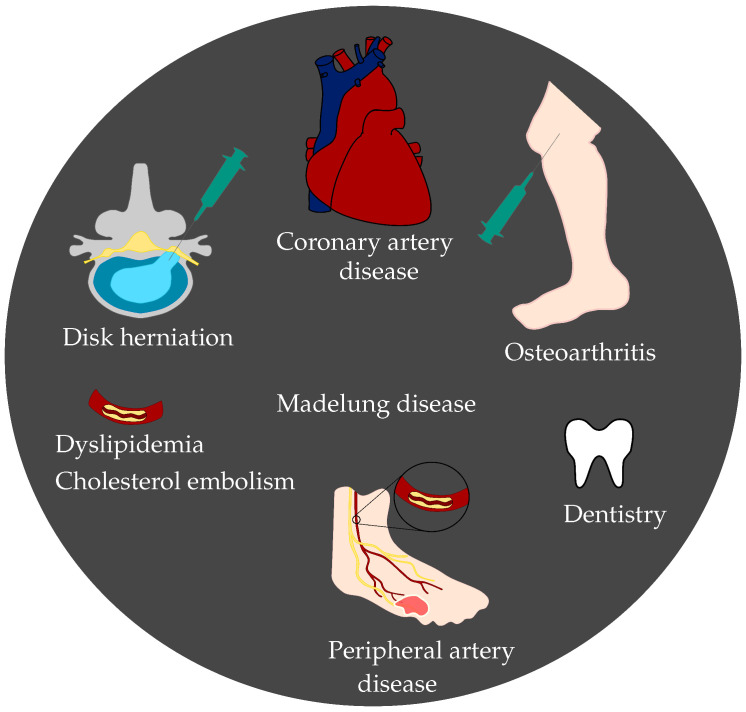
Schematic of possible applications for ozone treatment in healthcare. The applications reach from disk herniation and osteoarthritis, to coronary and peripheral artery disease, madelung disease, dyslipidemia, cholesterol embolism, and dentistry.

In contrast to this, ozone gas is already widely utilised for different treatments and health issues [5,9,10,11,12,13,14]. Exemplary scenarios are shown in Figure 1. Ozone is highly unstable and decays quickly into oxygen. Therefore, it has to be generated directly before the treatment. During the generation of ozone for medical applications, ultra-pure medical oxygen is utilised as input for the ozone generator. This leads to an output of an oxygen–ozone mixture for further utilisation. As ultra-pure medical oxygen instead of air is used as input for the ozone generator, humidity interference is not considered in detail when measuring ozone gas for medical applications.

One application of ozone is its utilisation as an effective alternative to pharmaceutical or surgical treatment of herniated disks [5,9]. Thereby, medical oxygen–ozone is applied with computer tomography (CT) guidance through an injection needle. This leads to chemonucleolysis of the intervertebral disk, and the inner part of the disk, the nucleus pulposus, reduces its volume progressively [9]. As a result, the pressure on the spinal nerve and the corresponding pain are released. For the treatment of knee osteoarthritis, oxygen–ozone is injected intra-articularly, which leads to long-term analgesic effects and increased mobility [14]. Furthermore, ozone has an anti-inflammatory effect, which is significant in both cases of intradiscal and intra-articular injections [14]. Another medical treatment, where ozone is applied, is the extracorporeal blood oxygenation and ozonation (EBOO) therapy. EBOO is a possible treatment for peripheral artery disease, coronary artery disease, dyslipidemia, madelung disease, and cholesterol embolism [10,11]. During the procedure, venous blood is taken from the patient and is enriched with oxygen–ozone. Afterwards, the blood is re-infused to the patient. In addition, ozone is applied in dentistry. One approach with good evidence is to utilise oxygen–ozone before the placement of dental sealants [12]. Another application is the utilisation of ozonated water for the treatment of avulsed teeth [13].

Thus, there is a wide variety of treatment procedures, for which it is essential that the ozone concentration can be monitored. Up to now, it has not been possible to measure the dissolved ozone concentration intracorporeal in human body liquids and tissues directly before and during treatment. For example, during EBOO procedures, the direct measurement of the ozone concentration is only possible before the ozonation process or by using indirect measurement through markers that are dependent to the ozone concentration [11]. Furthermore, there is no possibility of precise dosing and adjustment. This is particularly relevant for the treatment of herniated disks, because only relatively small amounts of oxygen–ozone (3–7 mL [9]) are required. Without monitoring the ozone concentration, health critical quantities might be applied, and contrariwise, too low a concentration of ozone can be ineffective. Therefore, it is important to integrate ozone sensors into medical and bioanalytical devices for the different treatment methods [16]. This enables automatic deactivation to avoid exceeding health critical limits. Furthermore, monitoring with a sensor allows one to get closer to the limit, which increases the therapeutic effect, without exceeding the limit value.

There are special requirements for a medical ozone sensor, which depend on the specific application. A fast response and recovery time is highly significant because of the half life of ozone in body liquids and tissues. Human tissues, apart from bones, typically have a high fraction of water. At a temperature of 40 °C, a pH value of 7, and an agitation speed of 100 rpm, ozone decomposes into water in about 25 min [17]. In comparison, ozone dissolves into blood within a few minutes [18]. The half life of ozone in body liquids and tissues is smaller than in water because biological organisms are well protected from strong oxidants, such as ozone, by antioxidant systems. Thereby, the antioxidants neutralise ozone and the radicals, which are formed by the reaction of ozone with body liquids and tissues. The implications of ozone on body liquids and tissues can also be analysed by considering the damage to deoxyribonucleic acid (DNA), amino acids, and lipids by ozone using measurements reported by Hepel et al. [19]. Therefore, sensors with a response time of several minutes cannot be utilised. For injection of oxygen–ozone in cases of herniated disks, the sensor response time has to be below 10–15 s, which is the duration of the treatment [9]. In addition, sensors that are in direct contact with blood should not be heated above 40 °C to avoid tissue damage. Since there are medical applications for ozone sensors without direct contact to body liquids and tissues, sensors with higher measurement temperatures are also included in this review. One example is the ozone therapy to treat a herniated disc. Here, a gas sensor can be utilised to monitor the concentration inside the injection needle during treatment. In addition, a medical application does not automatically imply that the sensor must be implanted in a human body. For example, medical temperature sensors typically measure body temperature outside the body. Furthermore, the sensor size has to be sufficiently small, in order to integrate the sensor into the injection needle. Moreover, performance of sensing elements is affected by bio-thiols, such as glutathione, which are in high concentration in body liquids and tissues. In blood, the concentration is in the range of 1–10 mM [20]. For electrochemical ozone sensors, the binding of exogenous bio-thiols to metal electrodes and several compound semiconductor electrodes is important because through the strong binding the surface of the electrodes is modified and reaction of ozone with the electrodes is limited. Stobiecka et al. [21] investigate the adsorption of bio-thiols on a gold surface and ligand replacement from a gold surface by glutathione.

**Figure 2:**
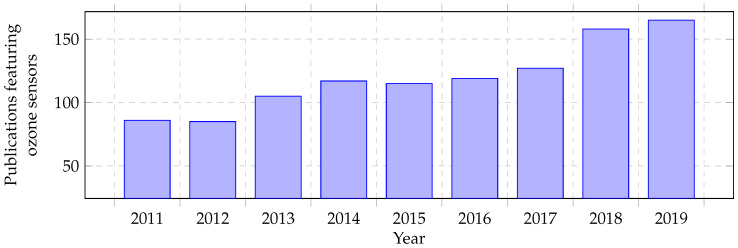
Number of publications featuring ozone sensors. Source: Scopus. Data extracted on 12 March 2020. All documents containing *ozone* and *sensor* were considered in the query and these documents were subdivided into the respective years.

As shown in Figure 2, there has been an increase of the number of publications during the last nine years (2011–2019) that are devoted to ozone sensors. While Figure 2 shows the overall trend, the following chapters cover the recent developments between 2016 and 2020.

The present review covers select recent works in ozone sensor technology in the period between 2016 and 2020. The sensors are subdivided into ozone gas and dissolved ozone sensors.

## 2. Measurement Principles for Ozone Sensing

**Figure 3:**
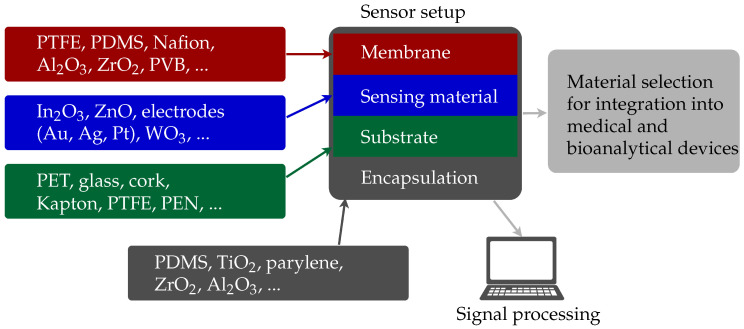
Schematic of a basic sensor structure, which consists of a membrane, sensing material, substrate, and encapsulation. The materials are employed in regard to integrate the sensor in medical and bioanalytical devices.

There are multiple measurement methods and sensing materials for sensor development. The basic sensor structure and materials utilised for the sensor setup are shown in Figure 3. The most important criterion for sensor development is the specific application with the corresponding requirements, which determines the sensor principle and the materials employed. The principles and materials are chosen to ensure the integration into medical and bioanalytical devices. In general, the sensing material is applied on a substrate. For a metal-oxide sensor, the sensitive area is, for example, based on an indium oxide thin film on a silicon substrate. In case of an amperometric sensor, the electrodes are, for example, printed with gold and silver nanoparticle inks. Furthermore, a membrane ensures selectivity; i.e., only substances that do not interfere with the desired measurement can get in contact with the sensing material. For sensors that are in contact with human body liquids and tissues, an encapsulation is needed to provide the sensors’ biocompatibility. It is essential that the measurement principle is not affected by the encapsulation. The encapsulation acts like a membrane and it is necessary to ensure that the substance intended for measurement can permeate through the encapsulation.

In the present review, the measurement methods are divided in electrochemical, optical, and volumetric. For electrochemical methods, amperometry and impedimetry are investigated. For optical methods, optical absorption, photoluminescence, and colorimetry are examined. Titration-based measurement methods are included in the category volumetric. In Table 1, an overview of the different measurement methods is given. The most important parameter for medical applications is the response time, as outlined in Section 1. A fast response time is highly significant because of the half life of ozone in water, body liquids, and tissues. Amperometric sensors have a response time between 15 s and 3 min. In contrast, the summarised impedimetric sensors provide a response time between 4 s and 15.5 min. Impedimetric sensors, which are based on carbon nanotubes as sensing material, have a high response time (15.5 min). Impedimetric metal-oxide sensors can only achieve a fast response time with light activation or high measurement temperatures. With a light activation the response time is in the range between 13 s and 12.9 min. Heated metal-oxide sensors can reach response times between 4 s and 10 min. Optical absorption sensors support, in many cases, real-time response. Reported photoluminescence sensors have response times between 10 and 20 min. The response time for colorimetric measurement methods is 2.5 min and for titration-based methods it is not reported.

Amperometric and optical absorption methods can be applied for dissolved ozone and ozone gas sensors. Impedimetric and photoluminescence methods can be employed for ozone gas sensors and titration and colorimetric methods for dissolved ozone measurements. Gas sensors can also be implemented for dissolved ozone measurement if a hydrophobic membrane is included. Furthermore, the gas stripping method can be used as a preprocessing step to transfer the measurement of dissolved ozone into a gas measurement.

### 2.1. Preprocessing Methods

#### 2.1.1. Gas Stripping

During gas stripping, the measurement of dissolved ozone is transferred into a gas measurement [22]. Therefore, ozone gas is separated from a liquid solution through mass transfer. A gas stream is guided over a liquid stream. The mass transfer between the surrounding gas and liquid solution depends on the relative concentration and the temperature of the liquid and is caused by the concentration difference between the gas and liquid solution. Afterwards, the gas concentration is measured by a gas sensor, for example, a photosensor or heated metal-oxide sensor, and the dissolved concentration of the liquid solution is calculated accordingly [23]. Advantageous for measurement in the gas phase compared to measurement of dissolved ozone is the absence of interference from particles and organic substances [22,23]. Therefore, sensors with an included gas stripping preprocessing step can also be used for non-pure water.

### 2.2. Electrochemical Measurement Methods

#### 2.2.1. Amperometry

**Figure 4:**
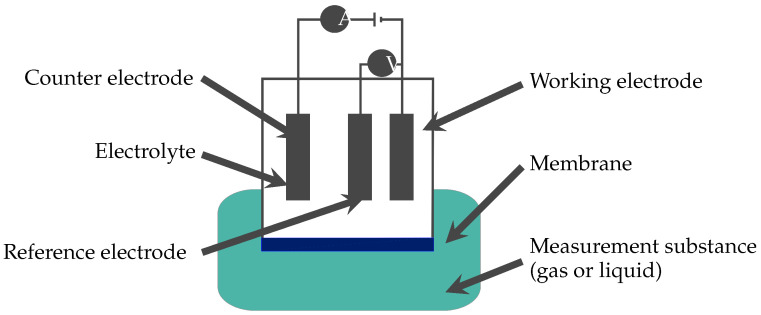
Schematic of an amperometric sensor. Between the working and reference electrode, a constant voltage is applied. The current, measured at the working electrode, changes when ozone is present in the measurement substance.

Amperometry is an electrochemical measurement method [24,25]. The basic sensor structure is shown in Figure 4 and consists of a working, counter, and reference electrode, electrolyte, and membrane. The sensor is surrounded by the measurement substance, which consists of a gas or liquid solution. Furthermore, the sensor is encapsulated by the combination of membrane material and the outer surface of the chamber around the electrolyte. A constant voltage between working and reference electrode is applied over time. Ozone permeates through the membrane and leads to a reduction reaction at the working electrode. The resulting current is measured at the working electrode. From the measured current value, the concentration is calculated.

As described in Table 2, a key parameter for amperometric sensing is the sensing material or unit, resulting in different measurement ranges, response times, and recovery times.

#### 2.2.2. Impedimetry

**Figure 5:**
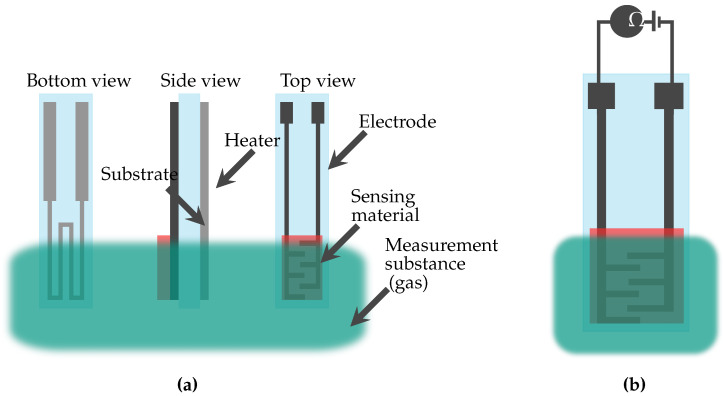
Schematic of an impedimetric sensor. (**a**) The bottom, side, and top views of the impedimetric sensor. (**b**) An expanded view of the sensor. Resistance Ω of the sensing material changes when ozone is present in the measurement substance.

Impedimetry, as amperometry, is an electrochemical measurement method [25]. Impedimetric sensors, as shown in Figure 5, consist of sensing material, substrate, electrodes, and a heat or light activation. They often include a heater which maintains a certain temperature of the sensing material. There are also sensors without a heater, which have a light activation that enables room temperature sensing. The light activation allows a decrease of the measurement temperature while maintaining a fast response time [26]. The sensor is surrounded by the measurement substance, which consists of gas. If a part of the electrodes that is not covered with the sensing material, can also get in contact with the measurement substance, this part has to be coated with a dielectric material.

Impedimetric sensors include resistive sensors for direct currents and impedance sensors for alternating currents. If the corresponding target substance is present, the value of the resistance or impedance of the sensing material changes. This value is measured and the concentration is calculated accordingly.

As reported in Table 2, key parameters for impedimetric sensing are the sensing material, electrode material, measurement temperature or wavelength in case of light activation, resulting in different measurement ranges, response times, and recovery times. For impedimetric sensors, the measurement temperature corresponds to the temperature of the sensing material. At room temperature T_Room_, the sensing material is not heated and the temperature is the same as the surrounding measurement substance.

### 2.3. Optical Measurement Methods

#### 2.3.1. Optical Absorption

**Figure 6:**
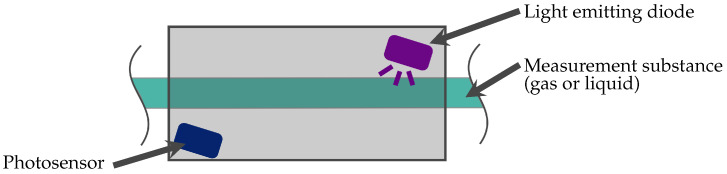
Schematic of an optical absorption sensor. The measurement method is based on measuring the light absorption of the measurement substance that is sent out by emitting diodes and detected by a photosensor.

The operating principle of optical absorption sensors, shown in Figure 6, is based on measuring the light absorption of a measurement substance sent out from a light source (for example light emitting diodes (LEDs)) and received by a detector (for example photosensors). This allows the measurement of characteristic absorption spectra for ozone gas or dissolved ozone. The absorption of ozone in the measurement substance is measured and compared to the absorption without ozone. Thereby, it is possible to calculate the concentration.

Key parameters are, as highlighted in Table 2, the sensing material or unit and measurement wavelength, resulting in different measurement ranges, response times, and recovery times.

Optical absorption sensors are mostly based on the Lambert-Beer law:

$$I\left(z\right)=I_{0}*e^{-\alpha *z}$$

Thereby, I_0_ represents the in-going and I(z) the out-going light, α the absorption coefficient and z the length of the light path in the absorbing medium. The absorption coefficient depends on the respective gas and the path length on the specific construction. Gengenbach and Sieber [27] present design rules and outline the significant factors for optical absorption gas sensor design. To ensure sensitivity, it is possible to either heat the absorption cell or increase the path length [27]. Furthermore, the optical path can be increased by folding the path by means of multiple reflections in the measuring chamber [27].

For optical absorption measurement of ozone, the most important variables are the absorption peaks. Ozone has an absorption peak at 254 nm and a second smaller one at 603 nm [28]. As mentioned in Section 1, an oxygen–ozone mixture is utilised for treatments with ozone. Therefore, the absorption of oxygen is also important in order to avoid any interference. The absorption peaks for oxygen are at 150, 688, and 762 nm [29,30]. Due to the well separated absorption spectra of ozone and oxygen and a respective selection of a suitable LED wavelength, interference of oxygen during ozone measurement can be prevented.

#### 2.3.2. Photoluminescence

Furthermore, there is the photoluminescence measurement method, based on optical methods, which was reported by Ando et al. [31]. The method is characterised through materials that change their optical properties, more specifically, the photoluminescence intensity, reversibly if ozone gas is present. These materials are called photoluminescence quantum dots. Thin films of core shell quantum dot particles are deposited on glass substrates and the photoluminescence intensity of the quantum dots is measured to calculate the ozone concentration. Ando et al. [31] stated that quantum dots of smaller size (and thus green-emitting) are more sensitive to ozone than quantum dots of larger size (and thus red-emitting). The quantum dot luminescence is only quenched by absorbed ozone, but not by pure oxygen, nitrogen, argon, and carbon dioxide. Thereby, selective measurement is enabled.

As described in Table 2, key parameters are the sensing material and measurement wavelength, resulting in different measurement ranges, response times, and recovery times.

#### 2.3.3. Colorimetry

Another optical measurement principle is the colorimetric measurement method. As the optical absorption measurement principle, colorimetry is also based on the Lambert-Beer law, reported in Section 2.3.1. Thereby, a detection reagent—such as N,N-diethyl-p-phenylenediamine (DPD) or DPD with potassium iodide—is added to ozonated water [32], which leads to a colour change. The absorption of the detection reagent in ozonated water is measured with the specific absorption spectrum that depends on the detection reagent. Adding potassium iodide in excess causes a growth of the sensor response [32].

As reported in Table 2, key parameters are the sensing material and wavelength of the photosensor, resulting in different measurement ranges, response times, and recovery times. This measurement principle cannot be applied for inline ozone measurements, but can for sensor calibration.

### 2.4. Volumetric Measurement Methods

#### 2.4.1. Titration

The concentration is determined by the volume of the titrant, which is added to the measurement substance until neutralisation occurs. Neutralisation means that the colour of the measurement substance changes visibly from clear to yellow-brown [33]. The concentration is measured through the volume of the titrant, for example, sodium thiosulfate, which is necessary for the neutralisation [33].

Key parameter is, as shown in Table 2, the titrant volume, represented by the category sensing unit, resulting in different measurement ranges. This method cannot be utilised as inline measurement principle. However, the method can be applied to calibrate ozone sensors.

### 2.5. Overview of the Key Parameters for the Ozone Measurement Methods

Table 2 shows an overview of the key parameters for amperometric, impedimetric, optical absorption, photoluminescence, colorimetric, and titration-based measurement methods. The sensing material or unit is a key parameter for all presented measurement methods, while the electrode material and measurement temperature is only for impedimetry a key parameter. The wavelength is a key parameter for impedimetry (in case of light activation), optical absorption, photoluminescence, and colorimetric measurement methods. For all presented measurement methods (except titration-based) the measurement range, response time, and recovery time are key parameters. For titration-based measurement methods, only the sensing unit and measurement range are key parameters.

In the following, sensor setups for the measurement of ozone in gases are described, followed by dissolved ozone.

## 3. Sensors for Measurement of Ozone Concentration in Gases

Selected publications of the period 2016–2020 for amperometric, impedimetric, optical absorption, and photoluminescence ozone gas sensors are listed in Table 3. Criteria distinguishing the measurement methods of the sensors are: sensing material, substrate, electrode material, measurement temperature, wavelength, measurement range, response time, recovery time, and commercial availability. Furthermore, repeatability, short-term and long-term drift, life expectancy, and maximum storage period are presented, if they are reported in the cited references. Additionally, influencing measurement parameters such as humidity and flow rate are provided.

### 3.1. Electrochemical Sensors

#### 3.1.1. Amperometric Sensors

For amperometric sensors, gold, silver, and platinum electrodes are utilised for sensing. Furthermore, polyethylene (PE), polyvinyl chloride (PVC), and polypropylene (PP) are commonly encountered as substrates. The measurement temperature is in the area between −30 and 90 °C. Described measurement ranges are between 0.23 and 5 × 10^4^ parts per billion (ppb). The lowest presented response and recovery time are 15 s and 180 s respectively. The majority of the presented sensors are commercially available. The reported flow rates are in the range between 0.3 and 0.35 L min^−1^ and humidity is between 10 to 90% relative humidity. Stated values for the repeatability are at zero ±7 ppb [35], at 40% of the range ±15% [35], for three independent sensor responses within ±1% of standard deviation [39], and ±0.4 ppb or 5% (the higher value of both) [41]. Short-term zero drift below 5 ppb per 24 h [35], short-term span drift below 1% full scale per 24 h [35], long-term zero drift below 10 ppb per month [35], long-term span drift below 2% full scale per month [35], and for long-term testing the failure rate below 1.3 failures per million hours of operation [38] are reported. Cited references in Table 3 present a life expectation of ten years [38], a life expectation of five to ten years [41], and a maximum storage period of six months [42].

#### 3.1.2. Impedimetric Sensors

The most common materials as sensing materials for impedimetric ozone gas sensors are zinc oxide, indium oxide, tungsten trioxide, and carbon nanotubes. For the sensing layer morphology, different nanostructures are utilised—such as nanocolumns [43], nanorods [26,45], platelets [46,48], nanothin films [44,59,64,68,69,70], nanoparticles [51,54,58,60,66], nanoislands [52], nanosheets [61], nanotubes [62,63], nanowires [66], and thick films [53]. As substrate, silicon dioxide/silicon, aluminium oxide, glass, aluminium, and quartz and for electrodes platinum, gold, titanium, and copper are commonly employed. The electrode thickness ranges between 100 [26,43,45,46,51,66,67] and 300 nm [61], while the distance between two opposing electrodes varies between 5 μm [64] and 50 mm [43,51,60]. The measurement temperature is between 0 and 350 °C. Some of the sensors utilise a light activation of the sensor surface that allows significant reduction of the response time at low measurement temperatures, which can be derived from Table 3. The wavelength of the light activation is mostly between 254 and 490 nm. Reported measurement ranges are between 0.5 and 1 × 10^6^ ppb. The lowest presented response and recovery time is 4 s respectively. The majority of the presented sensors are not commercially available. The flow rates are between 0.1 and 1 L min^−1^ and the reported humidity is in the range between 5 to 95% relative humidity. Investigated values for the repeatability range from 0.5 to 5.7 at 100 ppb per sensor minute value [47], show a correlation that is better than 0.7 [55], and is for sensor responses between ±10% of standard deviation [62]. For the short-term drift over three days, values between 2 and 4.7 ppb are reported [47].

Korotcenkov et al. [75] investigated the materials indium oxide and tin dioxide and the overall structural parameters that are essential for impedimetric ozone gas sensors. The authors state that the response time at 200 °C for a thin film thickness of 60 to 80 nm does not go beyond the value of 1 or 2 s. In general, the authors report that the amplitude of the sensor response is greater with a smaller film thickness and crystallize size and larger pore size.

An essential advantage of impedimetric ozone sensors is their high selectivity regarding ozone. In general, there are no membranes utilised to filter substances that do interfere with the desired measurement. Korotcenkov et al. [75] report on several significant factors that are necessary to achieve a high selectivity and sensitivity, such as:

- Compared to tin dioxide ozone sensors, indium oxide shows better results regarding ozone selectivity [76];
- Analysation of the sensor response by utilising a sensor array instead of a single sensor [77];
- Improvement of selectivity through surface modification or bulk doping [78].

### 3.2. Optical Sensors

#### 3.2.1. Optical Absorption Sensors

For optical absorption, sensing materials such as zinc oxide or methylene blue are applied on glass or quartz substrates. The measurement temperature complies with the room temperature. The wavelength of the sensors is between 190 and 800 nm. The measurement range of the presented sensors is between 3 and 700 ppb. Optical absorption sensors support mostly real-time sensing. Presented sensors are not commercially available. The flow rates are in the range between 0.05 and 2 L min^−1^ and humidity is between 50% and 90% relative humidity.

#### 3.2.2. Photoluminescence Sensors

For photoluminescence sensors, cadmium selenide-based core-shell type quantum dots can be employed as sensing material on glass substrates [31]. Measurement temperature is about 25 °C, the wavelength between 500 and 800 nm, and the measurement range between 100 and 5 × 10^5^ ppb. Furthermore, the response time is in the range between 10 to 20 min and the recovery time is one day. The reported sensor is not commercially available. The reported flow rate is 0.1 L min^−1^, while the relative humidity is not provided.

## 4. Sensors for Measurement of Dissolved Ozone Concentration

Table 4 provides information about selected recent works in the period between 2016 and 2020 for dissolved ozone sensors. The measurement methods range from amperometric and optical absorption to methods that are based on colour changes, such as colorimetric and titration-based methods. Thereby, the sensors are distinguished by the sensing unit or material, measurement temperature, wavelength, measurement range, response time, recovery time, and commercial availability. In addition, repeatability and zero drift are reported, if they are presented in the cited references. Furthermore, the flow rate is provided.

### 4.1. Electrochemical Sensors

#### 4.1.1. Amperometric Sensors

Reported amperometric sensors utilise gold, silver, platinum, and boron-doped diamond electrodes for sensing. Furthermore, measurement temperatures are between −5 and 50 °C and measurement ranges between 0.05 and 20 mg L^−1^. For response and recovery times, the lowest presented values are 15 s and 1 s respectively. Most of the reported sensors are commercially available. The flow rate ranges between 3.8 and 30 L h^−1^. Values for the repeatability are ±2% at a constant measurement temperature [82], below 1% repeatability and −1% stability per month without calibration [84], and 0.3% of the selected range or 0.01 parts per million (ppm) (the higher value of both) [85]. The reported zero drift is below 0.01 ppm per month [85].

In general, amperometric sensors include a membrane that filters other interfering substances. In case of ozonated water, oxygen needs to be filtered, as it is also an oxidizing substance that reacts at the working electrode. The main issue of membranes is that ozone needs to diffuse trough the membrane which increases the sensor’s response time. There are different possibilities for materials that can be utilised as a membrane which results in various ozone transfer rates and thereby response times. Jahnknecht et al. [92] report about ozone transfer rates for different membrane materials. The results are:

- For ceramic membranes made of aluminium oxide a transfer rate of 0.35 g m^−2^ h;
- For zirconium oxide 10 g m^−2^ h;
- For porous polytetrafluoroethylene (PTFE) 4 g m^−2^ h.

Zoumpouli et al. [93] stated that polydimethylsiloxane (PDMS), PTFE, and polyvinylidene difluoride (PVDF) have ozone mass transfer coefficients that are in the same order of magnitude. Furthermore, the authors suggest that an issue for other membranes, such as polyethersulfone (PES) and polyetherimide (PEI), is that they react with ozone, which leads to the decomposition of the membrane. Shanbhag et al. [94] point out that the permeability of ozone through PDMS is four times higher than the permeability of oxygen. Zhang et al. [95] compare the permeability of several gases through PDMS. The authors report that the permeability of oxygen is approximately twice as great as that of nitrogen. More details about the permeability of the PDMS membrane as a function of significant factors, such as thickness and area of the membrane, are also reported by Zhang et al. [95]. Investigated PDMS membranes by Shangbhag et al. [94] vary between 165 and 419 μm thickness and a permeation area between 6900 and 29,900 mm^2^.

Ishii et al. [80] and Einaga et al. [79] also report about membrane-free amperometric sensors with boron-doped diamond (BDD) material as the working electrode. The BDD film is deposited on silicon wafers and tungsten needles to fabricate microelectrodes, whereby the needles have a diameter of 20 μm [80]. BDD working electrodes are insensitive for reduction reactions with oxygen [80]. Therefore, selectivity can be achieved without using a membrane. For the counter electrode, platinum, and for the reference electrode, silver/silver chloride, were utilised. Thereby, neither electrolyte nor diaphragm is necessary because of the low background current, which decreases the voltage drop.

### 4.2. Optical Sensors

#### 4.2.1. Optical Absorption Sensors

For optical absorption sensors, photometers and fluorometers are utilised as sensing unit for dissolved ozone sensors. In general, the measurement temperature is between 5 and 40 °C. Applied wavelengths for the photometer are between 190 and 900 nm. For the fluorometer, the excitation spectra are between 213 and 335 nm and the emission spectra between 310 and 450 nm. Resulting measurement ranges are between 0.05 and 150 mg L^−1^. For optical absorption sensors, mostly real-time measurement is supported. Presented sensors are commercially available. Reported flow rates are up to 150 std L min^−1^ [91].

The most significant factors for measuring the ozone concentration with optical absorption methods are the absorption peaks. There are several options to utilise the peaks for measurement. Levine et al. [96] measure the ozone concentration in ozonated water with yellow and blue LEDs. These had wavelengths of approximately 584 (yellow) and 300 nm (blue), thereby taking into account that the LEDs bandwidths matching the ozone absorption peaks.

Furthermore, it is possible to measure the ozone concentration in blood plasma by using markers. Paolo et al. [11] suggest the utilisation of total antioxidant status (TAS), protein thiol groups (PTG), or thiobarbituric acid reactants (TBAR). Only the marker TAS can be measured directly with optical absorption methods and a photometer. The other two markers can be measured by colorimetric measurement methods and are explained in Section 4.2.3. With increasing ozone concentration in blood plasma, the TAS concentration decreases. Paolo et al. [11] investigated the optical absorption measurement of TAS at 600 nm.

#### 4.2.2. Optical Absorption with Gas Stripping Preprocessing Step

During the gas stripping process, ozone gas is separated from the liquid stream. Afterwards, the ozone gas concentration is determined. For the reported sensors, a photometer is applied as sensing unit to measure the ozone gas concentration. Alternatively, a heated metal-oxide sensor or other ozone gas sensor can be applied. The measurement temperature is 26.4 °C. For the utilisation of a photometer, the applied wavelength is 254 nm. Described measurement ranges are up to 100 mg L^−1^ and the lowest presented response and recovery times are 10 s respectively. Reported flow rates are approximately 0.3 L min^−1^. Reproducibility of these sensors is stated with 0.05 ppm or 1% of the reading (the higher value of both), while the zero drift is below 0.05 ppm per month [22].

#### 4.2.3. Colorimetric Sensors

The investigated colorimetric sensors are based on DPD or DPD and potassium iodide as the sensing unit for the detection reagent. The measurement temperature is 26.4 °C. Measurement ranges are ensured up to 5 mg L^−1^. The response time is about 2.5 min and the recovery time is not reported. The presented sensor is commercially available as a sensor unit.

As mentioned in Section 4.2.1, Paolo et al. [11] suggested the utilisation of makers to determine the ozone concentration in blood plasma. While only one of the markers can be determined directly with optical absorption methods, all of them, TAS, PTG, and TBAR, are measurable through colorimetric methods. With increasing ozone concentration in blood plasma, the TAS and PTG concentration decreases and TBAR increases. For colorimetric measurement, a detection reagent is added to the solution. After reaction of the ozone and blood plasma with the detection reagent, the marker concentration is measured quantitatively by observing the visible colour change with a photometer. Paolo et al. [11] investigate colorimetric measurement of the PTG concentration at 412 nm. Furthermore, Erel [97] reports colorimetric measurement of TAS at 660 nm. In addition, Kampa et al. [98] present colorimetric measurement of the TAS concentration at 450 nm.

### 4.3. Volumetric Sensors

#### Titration-Based Sensors

For titration-based sensors, one applicable titrant is sodium thiosulfate as the sensing reagent. As reported in Section 2.4.1, the concentration is determined through the volume of titrant that is added to the measurement substance until neutralisation, which is indicated by a colour change. The measurement temperature complies with the room temperature, while measurement ranges between 30 and 192 ppm are stated. Response and recovery times are not reported. Presented sensors are not commercially available as complete sensor units.

## 5. Fabrication Methods

**Figure 7:**
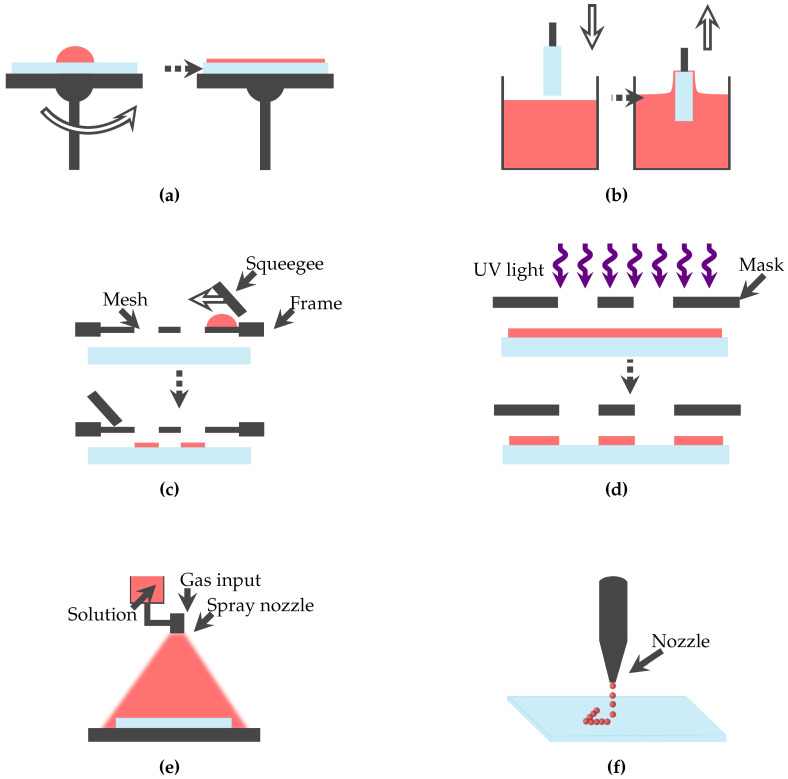
Schematic of typical fabrication methods for ozone sensors. The substrate is shown in blue and the applied ink or thin film material in red. (**a**) The spin-coating process. (**b**) A schematic of dip-coating/immersion. (**c**) The screen-printing process. (**d**) The UV photolithography process. (**e**) The spray-coating/spray pyrolysis process. (**f**) The inkjet-printing process.

Currently, the ozone sensors reported in Section 3 and Section 4 are manufactured by conventional fabrication methods, as presented in Table 5. The corresponding fabrication method and maximum process temperature are provided. The maximum process temperature is highly significant, because it restricts the utilisation of flexible substrates, as these can be damaged by high temperatures. Illustrations of typical fabrication methods are shown in Figure 7. Figure 7a–e shows conventional manufacturing methods of ozone sensors and Figure 7f a schematic of the inkjet-printing process.

Guth et al. [99] outline that for pH sensors—which have so far mainly been fabricated by precision engineering—thick film, thin film, and printing technologies on rigid planar substrates are technology routes for miniaturisation. These fabrication methods are also applied for the ozone sensors reported in Section 3 and Section 4 and summarised in Table 5. They are suitable for fabricating miniaturised structures, at elevated temperatures, however, on rigid substrates, such as aluminium oxide or silicon wafers. Sensing structure definition in these processes relies on masks, stencils, or lithography processes which require substrate planarity. Integration of a sensing element into a medical or bioanalytical device is an important requirement for increased application of ozone therapy, as outlined in Section 2. As this might require conformality to the instrument shape, the above fabrication technologies are not applicable.

Inkjet-printing, however, is a promising technology to fabricate miniaturised, conformal, and disposable sensors that can be integrated in medical and bioanalytical devices [101]. Inkjet-printing allows deposition of novel nanomaterials down to 50 μm structural resolution or below on polymer substrates. In order to exploit these benefits, materials (ink and substrate), printed structure design, printing process, printing system, and postprocessing must be carefully matched. For polymer substrates with low thermal stability, low temperature postprocessing routes, such as photonic sintering, have to be favoured. Gengenbach et al. [102] present a method for systematic development and verification of inkjet-printed multi-layer electronic circuits that are manufactured by applying a fully automated workflow from design to final printed system. In case flexibility of the printed structure is demanded by the application, adhesion between printed layer and substrate and layer morphology must be optimised [103].

In the following, inkjet-printed approaches for other electronic components that might be transferable to ozone sensors are reported. Thereby, the inkjet-printing technologies and methods are adaptable to realise an inkjet-printed ozone sensor. To the best of our knowledge, there are currently no inkjet-printed ozone sensors. So far, inkjet-printed ozone sensors have not been fabricated, because for the applications of the ozone sensors presented in this review, flexible substrates and conformal sensors are not necessary. In addition, the sensors can be fabricated in large quantities utilising established thick film and thin film processes. Inkjet-printing is promising for exploring new medical sensor applications, which are discussed in Section 1. For that purpose, fabrication of conformal medical sensors on flexible substrates is absolutely fundamental.

The ozone sensors presented in Section 3 and Section 4 are based on amperometric, impedimetric, optical absorption, photoluminescence, colorimetric, and titration-based measurement methods. Furthermore, gas stripping can be used as a preprocessing step.

Current approaches for inkjet-printed amperometric membrane-based oxygen or pH sensors can be modified, for example, through employing a membrane, and afterwards transferred for the utilisation as ozone sensor. They consist, for example, of inkjet-printed gold electrode arrays or working and counter electrodes made of gold nanoparticle ink and reference electrodes based on silver nanoparticle ink. Hu et al. [24] report an oxygen gas sensor based on gold electrode arrays that are inkjet-printed on a porous cellulose membrane. Furthermore, Xu et al. [104] present an inkjet-printed dissolved oxygen and pH sensor on a Kapton film, consisting of a three-electrode system. Another approach for an inkjet-printed dissolved oxygen sensor, investigated by Moya et al. [105], composes printed electrodes on a paper-based substrate (thickness: 65 μm and porosity: 80%). In addition, Moya et al. [106] report a stable fully inkjet-printed solid-state silver/silver chloride reference electrode, printed with four different materials—silver, SU-8, sodium hypochlorite, and polyvinyl butyral—on a polyethylene terephthalate (PET) substrate. In Section 4.1.1, PDMS is suggested as membrane material. Wu et al. [107] conceptualise inkjet-printed silver nanoparticles as microelectrodes on PDMS for microfluidic sensing. As outlined in Section 4.1.1, membrane-free approaches for amperometric sensors can be implemented with BDD working electrodes. Therefore, an impedimetric gas sensor, proposed by Laposa et al. [108], might be transferred. The authors report a sensor based on nanodiamond powder ink using the microwave linear antenna plasma enhanced chemical vapour deposition (MW-LA-PECVD) method for diamond growth. The sensor is fabricated by combining inkjet-printing, which was utilised for selective deposition of the ink on the electrodes, and the MW-LA-PECVD process. However, further research is still necessary to enable the boron-doping of the nanodiamond structure.

There are several approaches for inkjet-printed metal-oxide nanomaterials that can be adapted as impedimetric ozone sensors. Rieu et al. [109] and Kassem et al. [110] investigated inkjet-printed gas sensors, made of tin dioxide. Thereby, Rieu et al. [109] provided a fully inkjet-printed gas sensor on a polymer substrate to measure the carbon monoxide and nitrogen dioxide concentration. Furthermore, Kassem et al. [110] present a carbon monoxide gas sensor on polyimide foil that is sintered at 350 °C. In addition, Spinella et al. [111] conceptualised zinc-oxide-stacked multilayer gas sensors. Other approaches with inkjet-printed indium oxide semiconductor layers were described by Leppäniemi et al. [112], Hassan et al. [113], and Hong et al. [114]. Thereby, Leppäniemi et al. [112] optimised the inkjet-printing process of precursor solutions with indium oxide for thin film transistors, which are annealed at low temperatures (150 to 200 °C) and with application of UV exposure (160 nm). Furthermore, Hassan et al. [113] proposed the fabrication of a thin film transistor, also based on indium oxide nanoparticle ink, using laser ablation and inkjet-printing. Through laser ablation, the conductive ink channel resolution is improved, which results in smaller channel length. Hong et al. [114] investigated the humidity-sensing performance of a field effect transistor. The approaches for inkjet-printed metal-oxide precursor solution can be adapted for an inkjet-printed impedimetric ozone sensor. Another option for impedimetric ozone sensors is the carbon nanotube. Kim et al. [115] presented inkjet-printed single-walled carbon nanotubes (SWCNT) for thin film transistors.

Optical absorption sensors are based on the combination of light activation and photosensors. Tran et al. [116] investigated an all-inkjet-printed zinc oxide photosensor on Kapton substrate utilised as a wearable sensor at 370 nm with a response time of 0.3 s. Kaufhold et al. [117] reported an all-inkjet-printed photosensor that is based on silver electrode material and zinc oxide semiconductor material. The wavelength of the sensor is in the range between 310 and 395 nm, and the sensor is printed on a flexible polymer substrate. Furthermore, Nahlik et al. [118] presented an inkjet-printed photosensor based on zinc oxide and diamond precursor ink with the highest photoresponsivity at 365 nm. The authors state that the response time for a zinc oxide nanodiamond sensor is more than ten times higher than for a zinc oxide single-layered photosensor [118]. This supports the findings of Korotcenkov et al. [75], also reported in Section 3.1.2, which state that a sensor response optimisation can be achieved by adapting the sensing layer morphology. Another photosensor, published by Figueira et al. [119], comprises a fully printed photosensor with peak sensitivity at 302 nm on a cork sheet using zinc oxide/ethylcellulose ink. Additional inkjet-printed photosensors are described in a review by Zhan et al. [120]. Furthermore, the authors summarised in the review inkjet-printed LEDs and thus show possible solutions for optical absorption sensors.

For inkjet-printing of photoluminescence quantum dots, Han et al. [121] synthesised water-dispersible quantum dots and applied them to manufacture inkjet-printed photoluminescence images that can be read under UV light. Furthermore, the process was optimised by Pan et al. [122]. The authors inhibited the coffee-ring effect through employment of ethylene glycol with a high boiling point as solvent to disperse the quantum dots.

As colorimetric and titration-based sensors are not capable of inline measurements, the present review did not include inkjet-printed sensor approaches. However, in case of a colorimetric sensor, a detection reagent solved in the measurement substance can be measured with an optical absorption sensor. Thus, an inkjet-printed colorimetric sensor approach might be combined with a detection reagent that is applied through inkjet-printing, and an inkjet-printed optical absorption sensor.

Inkjet-printing approaches with a gas stripping preprocessing step can be combined through a device that separates the ozone gas from the liquid stream and an inkjet-printed gas sensor—such as amperometric, impedimetric, or optical absorption gas sensors.

## 6. Conclusions and Future Perspectives

In the present review, we have given an overview of selected recent developments in ozone sensor technology (2016–2020). Thereby, the reviews of David et al. [123,124] in 2015 about ozone gas sensors based on optical methods and Moya et al. [125] about inkjet-printed electrochemical sensors between 2015 and 2017 are supplemented and updated.

The focus in the present review is on gas and dissolved ozone sensors based on amperometric, impedimetric, and optical measurement methods. For the utilisation as a medical sensor, several requirements that depend on the specific applications are highly significant—such as measurement temperature, light activation, and response time. For example, sensors with high measurement temperatures and response times are often unsuitable for medical applications because they are in direct contact with body liquids and tissues, and the actual treatment can sometimes take only 10–15 s [9]. Furthermore, treating bodily tissues with UV light can be harmful. Sensors with reaction times of several minutes, high measurement temperatures, or UV light activation are not feasible and have to be optimised in this respect for the application in medical treatments.

Therefore, we have presented the most important requirements in tabular form. Information about sensing material or unit, substrate, electrode material, measurement temperature, light activation, measurement range, response and recovery time, and commercial availability is reported. Furthermore, fabrication methods and maximum process temperatures for the different sensors are provided. In addition, inkjet-printed fabrication approaches for other sensor applications that might be transferable to ozone sensors are presented. Inkjet-printing is a promising technology to decrease the sensor size and enable sensor integration in medical and bioanalytical devices. Kaufhold et al. [117] report that printing of functional inks is a fast growing market. Further research is still necessary in order to develop, optimise, and integrate the sensors. Especially for integrating sensors in medical devices, a detailed analysis of the available space for the specific medical application as well as current state of the art sensor size are essential. 

## Figures and Tables

**Table 1:** Overview of the measurement methods for ozone sensing. Information about the ranges for several criteria, such as measurement temperature and response time, for the different measurement methods is provided.

Measurement Method	Sensing Unit	Ozone (Dissolved/Gas)	T_Measurement_ [°C]	t_Response_	Range of Typical Wavelengths
amperometric	electrodes	gas	−30 to 90	15 s to 3 min	NA
	electrodes	dissolved	−5 to 50	15 s to 3 min	NA
impedimetric	metal-oxide	gas	0 to 350	4 s to 10 min	NA
	metal-oxide	gas	25 to 26	13 s to 12.9 min	light activation ^a^
	nanotubes	gas	25 to 75	15.5 min	NA
opticalabsorption	photosensor	gas	T_Room_	real-time	190 to 800 nm
	photosensor	dissolved	5 to 40	real-time	190 to 900 nm
photoluminescence	photosensor	gas	25	10 to 20 min	500 to 800 nm
colorimetric	detection reagent and photosensor	dissolved	T_Room_	2.5 min	NR
titration	titrant	dissolved	T_Room_	NR	NA

NR: not reported; NA: not applicable. ^a^ In case of an impedimetric metal-oxide sensor, the light activation of the sensor surface allows a significant reduction of the response time at low measurement temperatures.

**Table 2:** Overview of the key parameters for amperometric, impedimetric, optical absorption, photoluminescence, colorimetric, and titration-based measurement methods.

Measurement Method	Sensing Material/Unit	Electrode Material	T_Measurement_	λ	Measurement Range	t_Response_	t_Recovery_
amperometric	✓	✗	✗	✗	✓	✓	✓
impedimetric	✓	✓	✓	✓	✓	✓	✓
optical absorption	✓	✗	✗	✓	✓	✓	✓
photoluminescence	✓	✗	✗	✓	✓	✓	✓
colorimetric	✓	✗	✗	✓	✓	✓	✓
titrtration	✓	✗	✗	✗	✓	✗	✗

**Table 3:** A summary of important publications in the period between 2016 and 2020 focused on amperometric, impedimetric, optical absorption, and photoluminescence ozone gas sensors. Information about highly significant criteria—such as sensing material, measurement temperature, and response time—is provided. For impedimetric sensors, an entry of the wavelength shows that light activation of the sensor surface is present, which enhances the sensor response at low temperatures. In cases of optical absorption and photoluminescence sensors, the wavelength is necessary for the optical measurement.

Measurement Method	Sensing Material	Substrate	Electrode Material	T_Measurement_ [°C]	λ [nm]	Measurement Range [ppb]^a^	t_Response_ (c_Ozone_ [ppb])	t_Recovery_	Com. av.	Year [Ref.]
amperometric	WE: NR; RE: NR; CE: NR	NR	NA	20	-	20 to 250	<180 s (NR)	<180 s	yes	2016 [34,35]
	WE: NR; RE: NR; CE: NR	NR	NA	12 to 26	-	5 to 1 × 10^4^	NR	NR	yes	2017 [36]
	WE: NR; RE: NR; CE: NR	NR	NA	−30 to 50	-	20 to 2 × 10^4^	15 s (NR)	NR	yes	2017 [37,38]
	WE: Au; RE: Ag; CE: Pt	silicon	NA	25, 35, 50	-	0.23 to 180	NR	NR	no	2017 [39]
	WE: Au, Ag, Pt; RE: Au,	PE, PVC,	NA	15 to 90	-	NR	NR	NR	no	2018 [40]
	Ag, Pt; CE: Au, Ag, Pt	PP								
	WE: Au; RE: NR; CE: Ag	NR	NA	−5 to 45	-	0.6 to 5 × 10^4^	30 s (NR)	NR	yes	2020 [41]
	WE: NR; RE: NR; CE: NR	NR	NA	−20 to 50	-	0 to 5 × 10^3^	NR	NR	yes	2020 [42]
impedimetric	ZnO	SiO_2_/Si	Pt	200	-	100 to 1 × 10^6^	9.6 s (100)	45.6 s	no	2016 [43]
	Co_x_Zn_1−x_O	SiO_2_/Si	Pt	150 to 350	-	42 to 560	40 s (NR)	6 min	no	2016 [44]
	ZnO mod. NiPc	SiO_2_/Si	Pt	250	-	80 to 890	22 s (80)	33 s	no	2016 [45]
	NiCo_2_O_4_	SiO_2_/Si	Pt	200	-	28 to 165	32 s (28)	60 s	no	2016 [46]
	NR	NR	NR	12 to 32	-	1.5 to 110	10 min (90)	NR	yes	2016 [47]
	NR	NR	NR	12 to 32	-	0.5 to 110	5 min (90)	NR	yes	2016 [47]
	WO_3_	NR	NR	12 to 32	-	1 to 110	10 min (90)	NR	no	2016 [47]
	NiAl-LDH	Al	Au	25	-	15 to 3580	4 s (15)	4 s	no	2017 [48]
	NR	NR	NR	0 to 40	-	1 to 150	NR	NR	yes	2017 [49,50]
	ZnO-SnO_2_	Al_2_O_3_	Pt	26	325	20 to 300	13 s (60)	90 s	no	2017 [51]
	Ag (APTMS)	glass	NR	NR	-	15 × 10^4^ to 1 × 10^6^	50 s (2 × 10^5^)	NR	no	2017 [52]
	Ag (PVA)	glass	NR	NR	-	18 × 10^4^ to 1 × 10^6^	15 s (2 × 10^5^)	NR	no	2017 [52]
	Au (APTMS)	glass	NR	NR	-	15 × 10^4^ to 1 × 10^6^	70 s (2 × 10^5^)	NR	no	2017 [52]
	Au (PVA)	glass	NR	NR	-	18 × 10^4^ to 1 × 10^6^	25 s (2 × 10^5^)	NR	no	2017 [52]
	In_2_O_3_ dop. WO_3_	Al_2_O_3_	Pt	75	-	200 to 500	60 s (200)	60 to 120 s	no	2017 [53]
	ZnO	Si/SiO_2_	Ti/Pt	25	390	35 to 165	NR	NR	no	2017 [54]
	NR	NR	NR	25	-	10 to 1000	NR	NR	yes	2017 [55,56,57]
	WO_3_	Al	Au	150	-	500 to 2000	NR	NR	no	2017 [58]
	am.-IGZO	glass	NR	25	365	500 to 5000	775 s (500)	2470 s	no	2018 [59]
	CuWO_4_	SiO_2_/Si	Pt	200 to 290	-	15 to 1400	7 s (90)	5 to 10 s	no	2018 [60]
	ZnO	Al	Au	300	-	NR to 100	NR	NR	no	2018 [61]
	CNT func. ODA	Al_2_O_3_	Pt	75	-	200 to 500	15.5 min (200)	28.7 min	no	2018 [62]
	CNT	FR-4	Cu	T_Room_	-	200 to 500	NR	NR	no	2018 [63]
	CH_3_NH_3_Pbl3_−x_Cl_x_	glass	Pt	T_Room_	-	5 to 2500	225 s (180)	40 to 60 s	no	2018 [64]
	TiO_2_-In_2_O_3_	Al	Au	25	405	40 to 2000	40 s (2000)	280 s	no	2018 [65]
	V_2_O_5_/TiO_2_	SiO_2_/Si	Pt	300	-	90 to 1250	4.4 min (1250)	5 to 16 min	no	2019 [66]
	ZnO mod. Au	BOPET	Pt	26	370	30 to 570	13 s (30)	29 s	no	2019 [26]
	Ag-TiO_2_	glass	Au	T_Room_	UV, blue	100	NR	NR	no	2019 [67]
	Zn_0.95_Co_0.05_O	SiO_2_/Si	Pt	250	-	20 to 1040	40 s (260)	100 s	no	2019 [68]
	IGZO-dec.	quartz	ITO	T_Room_	254	NR	NR	NR	no	2020 [69]
	am.-Ga_2_O_3_									
	rGO/WO_3_	quartz	NR	T_Room_	-	100 to 1000	NR	NR	no	2020 [70]
optical absorption	KI and α-CD	glass	NA	20 to 22	320 to 750	3 to 150	NR	NR	no	2017 [71]
	ZnO or LiGaO_2_	NR	NA	NR	250 to 290	NR	NR	NR	no	2017 [72]
	rGO/ZnO	quartz	NA	T_Room_	190 to 500	300 to 700	real-time	real-time	no	2018 [73]
	methylene blue	quartz	NA	23	400 to 800	10 to 200 ppbv	real-time	real-time	no	2019 [74]
photo-luminescence	QD CdSe	glass	NA	25	500 to 800	100 to 5 × 10^5^	10 to 20 min (NR)	1 day	no	2016 [31]

NR: not reported; NA: not applicable; ^a^ unless otherwise stated; BOPET: bi-axially oriented poly(ethylene terephthalate); NiPc: nickel phthalocyanine; CNT: carbon nanotubes; mod.: modified by; IGZO: indium gallium zinc oxide; Ref.: references; NiAl-LDH: nickel aluminide layered double hydroxide; am.: amorphous; ITO: tin-doped indium oxide; rGO: reduced graphene oxide; PANI: polyaniline nanostructures; KI: potassium iodide; dec.: decorated; dop.: doped with; func.: functionalised by; ODA: octadecylamine groups; FR-4: glass-reinforced epoxy laminate material; QD CdSe: cadmium selenide based core-shell type quantum dots (CdSe/CdZnS, CdSe/ZnS, and CdSeTe/ZnS); α-CD: α-cyclodextrin; APTMS: aminopropyl trimethoxysilane; PVA: polyvinyl alcohol; Com. av.: commercially available; WE: working electrode; RE: reference electrode; CE: counter electrode.

**Table 4:** An overview of dissolved ozone sensors in the period between 2016 and 2020. Highly significant criteria—such as sensing unit, measurement temperature, and response time—are provided.

Measurement Method	Sensing Unit/ Sensing Material	T_Measurement_ [°C]	λ [nm]	Measurement Range	t_Response_	t_Recovery_	Commercially Available	Year [Ref.]
amperometric	WE: BDD; RE: Pt; CE: Pt	25	-	0.185 to 740 μM	NR	NR	no	2017 [79,80]
	WE: Au; RE: Au; CE: NR	26.4	-	0 to 5 mg L^−1^	NR	1 s	yes	2020 [32,81]
	WE: Au; RE: NR; CE: NR	26.4	-	0 to 3 mg L^−1^	30 s	1 s	yes	2020 [32,82]
	WE: NR; RE: NR; CE: NR	0 to 45	-	0.05 to 20 mg L^−1^	15 s	NR	yes	2020 [83]
	WE: Au; RE: NR; CE: Ag	0 to 45	-	0 to 20 mg L^−1^	50 s	NR	yes	2020 [84]
	WE: NR; RE: NR; CE: NR	0 to 50	-	0 to 200 ppb	60 s	NR	yes	2020 [85]
	WE: Au; RE: Ag; CE: Ag	−5 to 50	-	0 to 10 ppm	90 s	NR	yes	2020 [86]
	WE: NR; RE: NR; CE: NR	5 to 50	-	0 to 5 mg L^−1^	30 s	NR	yes	2020 [87]
	WE: NR; RE: NR; CE: NR	0 to 40	-	0 to 5 mg L^−1^	3 min	NR	yes	2020 [88]
optical absorption	photometer	25	190 to 900	0.05 0 to 9 mg L^−1^	real-time	real-time	yes	2016 [89]
	fluorometer	15	excitation: 213 to 335 emission: 310 to 450	0 to 5 mg L^−1^	real-time	real-time	yes	2017 [90]
	photometer	5 to 40	NR	0 to 150 mg L^−1^	2 s	NR	yes	2020 [91]
	photometer (preprocessing: gas stripping)	NR	254	0 to 890 ppb	10 s	NR	no	2016 [23]
	photometer (preprocessing: gas stripping)	26.4	254	0 to 100 mg L^−1^	20 s	10 s	yes	2020 [22,32]
colorimetric	DPD or DPD with KI	26.4	NR	0 to 5 mg L^−1^	2.5 min	NR	yes	2020 [32]
titration	Na_2_S_2_O_3_	T_Room_	-	30 to 192 ppm	NR	NR	no	2019 [33]

NR: not reported; WE: working electrode; RE: reference electrode; CE: counter electrode; BDD: boron-doped diamond electrode; DPD: N,N-diethyl-p-phenylenediamine; KI: potassium iodide.

**Table 5:** Overview of fabrication methods for ozone sensors. Information about the measurement method, sensing material, fabrication method, and maximum process temperature is provided.

Measurement Method	Sensing Material	Fabrication Method	T_Process,max_ [°C]	Year [Ref.]
amperometric (gas)	WE: NR; RE: NR; CE: NR	screen-printing	NR	2017 [37,38]
	KI-PANI	electropolymerisation	100	2017 [39]
amperometric (dissolved)	WE: BDD; RE: Pt; CE: Pt	MPCVD	NR	2017 [79,80]
impedimetric (gas)	ZnO	microwave-assisted hydrothermal synthesis	120	2016 [43]
	Co_x_Zn_1−x_O	polymeric precursor spin-coating	500	2016 [44]
	ZnO mod. NiPc	hydrothermal synthesis and dipping	95	2016 [45]
	NiCo_2_O_4_	urea-assisted chemical co-precipitation [100]	450	2016 [46]
	NiAl-LDH	hydrothermal synthesis and dip-coating	100	2017 [48]
	ZnO_S_nO_2_	hydrothermal synthesis	200	2017 [51]
	Au and Ag	immersion, dipping, or spin-coating	160	2017 [52]
	In_2_O_3_ dop. WO_3_	screen-printing	600	2017 [53]
	ZnO	magnetron sputtering and spin-coating	80	2017 [54]
	WO_3_	liquid precursor flame spraying	200	2017 [58]
	am.-IGZO	RF sputtering	100	2018 [59]
	CuWO_4_	sono-chemical route	500	2018 [60]
	ZnO	electron beam evaporation and hydrothermal synthesis	100	2018 [61]
	CNT func. ODA	spray-coating and screen-printing	100	2018 [62]
	CNT	spray-coating	NR	2018 [63]
	CH_3_NH_3_Pbl_3−x_Cl_x_	spin-coating	100	2018 [64]
	TiO_2_-In_2_O_3_	dip-coating	400	2018 [65]
	V_2_O_5_/TiO_2_	hydrothermal synthesis	500	2019 [66]
	ZnO mod. Au	photolithography, hydrothermal synthesis, and thermal evaporation (gold deposition in vacuum)	95	2019 [26]
	Zn_0.95_Co_0.05_O	spray pyrolysis	300	2019 [68]
	IGZO-dec.	UV photolithography and	T_Room_	2020 [69]
	am.-Ga_2_O_3_	RF magnetron sputtering		
	rGo/WO_3_	hydrothermal synthesis and dip-coating	180	2020 [70]
optical absorption (gas)	KI and α-CD	immersion	NR	2017 [71]
	rGO/ZnO	ultra-sonic assisted solution process and immersion	400	2018 [73]
	methylene blue	dip-coating	450	2019 [74]
	Ag-TiO_2_	RF magnetron sputtering	250	2019 [67]
photoluminescence (gas)	QD CdSe	cast deposition	NR	2016 [31]

Ref.: references; NR: not reported; mod.: modified by; NiPc: nickel phthalocyanine; NiAl-LDH: nickel aluminide layered double hydroxide; dop.: doped with; PANI: polyaniline nanostructures; KI: potassium iodide; am.: amorphous; CNT: carbon nanotubes; IGZO: indium gallium zinc oxide; rGO: reduced graphene oxide; dec.: decorated; RF: radio frequency; func.: functionalised by; ODA: octadecylamine groups; QD CdSe: cadmium selenide based core-shell type quantum dots (CdSe/CdZnS, CdSe/ZnS, and CdSeTe/ZnS); α-CD: α-cyclodextrin; MPCVD: microwave plasma-assisted chemical vapour deposition process; WE: working electrode; RE: reference electrode; CE: counter electrode; BDD: boron-doped diamond electrode.

## Abbreviations

The following abbreviations are used in this manuscript:

BDD	boron-doped diamond
CT	computer tomography
DNA	deoxyribonucleic acid
DPD	N,N-diethyl-p-phenylenediamine
EBOO	extracorporeal blood oxygenation and ozonation
LEDs	light emitting diodes
MW-LA-PECVD	microwave linear antenna plasma enhanced chemical vapour deposition
ppb	parts per billion
ppm	parts per million
PDMS	polydimethylsiloxane
PEI	polyetherimide
PES	polyethersulfone
PE	polyethylene
PEN	polyethylene naphthalate
PET	polyethylene terephthalate
PP	polypropylene
PTFE	polytetrafluoroethylene
PVC	polyvinyl chloride
PVDF	polyvinylidene difluoride
PTG	protein thiol groups
SWCNT	single-walled carbon nanotubes
TAS	total antioxidant status
TBAR	thiobarbituric acid reactants
